# Imatinib in combination with phosphoinositol kinase inhibitor buparlisib in patients with gastrointestinal stromal tumour who failed prior therapy with imatinib and sunitinib: a Phase 1b, multicentre study

**DOI:** 10.1038/s41416-020-0769-y

**Published:** 2020-03-09

**Authors:** Hans Gelderblom, Robin L. Jones, Suzanne George, Claudia Valverde Morales, Charlotte Benson, Jean-Yves Blay, Daniel J. Renouf, Toshihiko Doi, Axel Le Cesne, Michael Leahy, Sabine Hertle, Paola Aimone, Ulrike Brandt, Patrick Schӧffski

**Affiliations:** 1 Leiden University Medical Center, Leiden, The Netherlands; 2 The Royal Marsden Hospital and Institute of Cancer Research, London, UK; 3 Dana-Farber Cancer Institute Boston, Boston, MA USA; 4 Vall D’Hebron University Hospital, Barcelona, Spain; 5 The Royal Marsden Hospital, London, UK; 6 Centre Léon Bérard, Lyon, France; 7 Unicancer, Paris, France; 8 BC Cancer - Vancouver Centre, Vancouver, Canada; 9 National Cancer Center Hospital East, Kashiwa, Japan; 10 Institut Gustave Roussy, Villejuif Cedex, France; 11 The Christie Hospital, Manchester, UK; 12 Novartis Pharma AG, Basel, Switzerland; 13 University Hospitals Leuven, Department of General Medical Oncology, Leuven Cancer Institute, KU Leuven, Leuven, Belgium

**Keywords:** Gastric cancer, Gastric cancer

## Abstract

**Background:**

The majority of patients with advanced gastrointestinal stromal tumours (GISTs) develop resistance to imatinib and sunitinib, the standard of care for these patients. This study evaluated the combination of buparlisib, an oral phosphoinositide 3-kinase (PI3K) inhibitor, with imatinib in patients with advanced GIST, who have failed prior therapy with imatinib and sunitinib.

**Methods:**

This Phase 1b, multicentre, open-label study aimed to determine the maximum tolerated dose (MTD) and/or a recommended Phase 2 dose of buparlisib in combination with 400 mg of imatinib through a dose-escalation part and a dose-expansion part, and also evaluated the clinical profile of the combination.

**Results:**

Sixty patients were enrolled, including 25 in the dose-escalation part and 35 in the dose-expansion part. In the combination, MTD of buparlisib was established as 80 mg. No partial or complete responses were observed. The estimated median progression-free survival was 3.5 months in the expansion phase. Overall, 98.3% of patients had treatment-related adverse events (AEs), including 45% with grade 3 or 4 AEs.

**Conclusions:**

Buparlisib in combination with imatinib provided no additional benefit compared with currently available therapies. Due to the lack of objective responses, further development of this combination was not pursued for third-line/fourth-line advanced/metastatic GIST.

**Trial registration number:**

NCT01468688.

## Background

Gastrointestinal stromal tumours (GISTs) are rare tumours of mesenchymal origin, most likely arising from precursors of the interstitial cells of Cajal of the gastrointestinal tract.^1^ The estimated incidence of GIST is 12–15 cases per 1 million population in Western countries.^2,3^ Approximately 75–80% of GIST cases show activating mutations in *KIT* (CD117). The most common *KIT* mutations are found in exon 11 in ~70% of cases, while *KIT* exon 9 mutations are relatively less common and are found in 10–12% of GIST cases. Mutations in *KIT* exons 13 and 17 are rare.^4^ A subset (5–8%) of GIST harbours *PDGFRA* mutations without the *KIT* mutations.^5,6^ The first-line treatment for patients with primary GIST is surgical resection. However, with surgery alone, the recurrence rates are more than 50% within 2 years, and the 5-year disease-specific survival rate is about 54%.^7^

Imatinib (Gleevec®/Glivec®, Novartis), a tyrosine kinase inhibitor (TKI), is approved for the treatment of adult patients with *KI*T^+^ (CD117) unresectable/metastatic GIST, and as adjuvant treatment following resection of high-risk GIST.^8,9^ For patients who develop either primary or secondary resistance to imatinib due to mutations in *KIT* or *PDGFRA*, sunitinib, a TKI-targeting multiple tyrosine kinase, demonstrated a significant improvement in progression-free survival (PFS),^10^ and is globally approved for the treatment of metastatic GIST.^11^ Following the failure of imatinib and sunitinib, treatment with regorafenib, another TKI targeting multiple oncogenic pathways, showed a significant improvement in PFS when compared with placebo, and was approved as a third-line therapy in 2013.^9,12,13^ In addition, reintroduction of imatinib was found to be marginally beneficial in patients with metastatic GIST in delaying disease progression.^14,15^ Despite the availability of current treatment options, there still remains a high unmet medical need for patients with advanced/metastatic GIST.

Advanced GISTs are commonly associated with resistance against approved TKIs, perhaps due to the gain of TKI-resistant mutations in *KIT*.^16^ In addition, resistance might be the result of either genomic amplification of *KIT* or activation of alternative oncogenic signalling mechanisms, including the phosphoinositide 3-kinase (PI3K)/protein kinase B (AKT)/mammalian target of rapamycin (mTOR) pathway.^16^ The PI3K/AKT signalling pathway has also been shown to be activated in a KIT-dependent manner in primary GISTs, specifically with mutations in exons 9, 11 and 13.^17^ Buparlisib (BKM120), an oral PI3K inhibitor, has shown antitumour activity against both imatinib-sensitive and imatinib-resistant cell lines with activated PI3K pathway. Buparlisib in combination with imatinib, has shown a greater suppression of tumour growth compared with single-agent imatinib or buparlisib. In patient-derived GIST xenograft models with imatinib-resistant cell lines, similar results were observed, with a significant inhibition of the tumour growth with the buparlisib and imatinib combination compared with single agents.^18–20^

It was thus hypothesised that the inhibition of PI3K/AKT signalling pathways by buparlisib in addition to continuous inhibition of *KIT*/*PDGFRA* mutations by imatinib could lead to increased clinical efficacy, and possibly overcome the acquired resistance to imatinib. This Phase 1b study assessed the maximum tolerated dose (MTD), and the safety and efficacy of the combination of buparlisib and imatinib in patients with advanced GIST, who had failed both imatinib and sunitinib.

## Methods

### Patients

Adult patients with histologically confirmed unresectable or metastatic GIST who had failed prior therapy with both imatinib and sunitinib were enrolled. Patients were required to have at least one measurable lesion as defined by Response Evaluation Criteria in Solid Tumors (RECIST, version 1.1), a World Health Organisation performance status of 0–2 and adequate bone marrow and organ function. For the dose-expansion part, radiological confirmation of disease progression during previous therapy was required, and up to three lines of prior therapy were permitted. Patients with previous treatment with PI3K inhibitors, any ongoing condition of Common Terminology Criteria for Adverse Events (CTCAE) grade ≥2 related to previous imatinib and/or sunitinib and poorly controlled diabetes mellitus (defined as glycosylated haemoglobin >8%), were excluded from the study. Since buparlisib is known to be associated with psychiatric disorders, including mood disorders, anxiety and depression, patients with a medically documented history of active major psychiatric disorders were not eligible. At screening, patients who had a cut-off score of ≥10 in the 9-item depression scale of the Patient Health Questionnaire-9 (PHQ-9) or a cut-off score of ≥15 in the Generalized Anxiety Disorder Assessment-7 (GAD-7) mood scale, or selected a positive response to question numbers 1, 2 and 3 to question number 9 regarding potential for suicidal thoughts or ideation in the PHQ-9 (independent of the total score of the PHQ-9) were excluded from the study.^21,22^

### Study design and treatment

This was a multicentre, open-label, Phase 1b study to investigate the safety and tolerability of escalating doses of buparlisib in combination with imatinib in patients with metastatic/unresectable GIST. The primary end point was to determine the MTD and/or a recommended Phase 2 dose (RP2D) of buparlisib with 400 mg of imatinib. The key secondary end points were to assess the safety, tolerability and clinical activity of the combination.

The study was conducted in two stages: a dose-escalation part to establish the MTD and/or RP2D and a dose-expansion part at the achieved MTD/RP2D. In the dose-escalation part, successive cohorts of at least three patients received increasing doses of buparlisib. The initial dose was 40 mg once daily, which was 40% of 100 mg/day (MTD as monotherapy), followed by 50, 70, 80 and 100 mg of daily doses along with imatinib, 400 mg daily, until the determination of MTD and/or RP2D of buparlisib. At least six patients must have been treated with the specific dose prior to declaring it as MTD. In the dose-expansion part, patients received either imatinib (arm 1) or buparlisib (arm 2) from days 1 to 8 (monotherapy run-in phase), followed by the combination therapy on day 9, or patients were treated with the combination therapy starting on day 1 (arm 3). All patients were treated until disease progression or early discontinuation.

This study was reviewed and approved by the independent ethics committee and/or local review board at each participating institution, and conducted according to the ethical principles described in the Declaration of Helsinki. All participants provided written informed consent prior to the study.

### Assessments

#### Efficacy

The tumour response was determined locally by the investigator at each site according to RECIST version 1.1. The efficacy assessments included clinical benefit rate (CBR), overall response rate (ORR), disease control rate (DCR) and PFS. The CBR was defined as the proportion of patients with the best overall response of complete response (CR) or partial response (PR), or a response of stable disease (SD) that lasted for ≥16 weeks after treatment initiation; ORR was defined as the proportion of patients with the best overall response of CR or PR. The DCR was the proportion of patients with the best overall response of CR or PR or SD. The PFS was defined as the time from the date of the first study treatment to the date of the first documented disease progression or date of death due to any cause, whichever occurred first. The response was assessed using computed tomography or magnetic resonance imaging at screening on day 1 of cycle 3, on day 1 of every second cycle through cycle 11, every 3 months after cycle 12 and every 6 months after cycle 24. The same technique, whether computed tomography or magnetic resonance imaging, that was used to assess the response at screening for a patient, was used throughout the study for a particular patient.

#### Safety

The adverse events (AEs) were coded using the Medical Dictionary for Regulatory Activities (MedDRA) version 19.0, while the severity was graded according to the CTCAE version 4.03. The safety assessments were performed at each visit. Patients were also asked to complete the mood scales for depression (PHQ-9) and anxiety (GAD-7) at screening, twice during cycle 1 and once during each subsequent cycle, including at the end of treatment. From cycle 12, the questionnaires were completed every 3 months, and after cycle 24, every 6 months.

### Statistical methods

The full-analysis set (FAS) and safety set consisted of all patients who received at least one dose of buparlisib or imatinib. The FAS was used for summarising the baseline and demographic characteristics. The efficacy analysis focused on patients enrolled in the dose-expansion part in the FAS. The Kaplan–Meier method was used for estimating PFS. The ORR, DCR and CBR were summarised with their 95% confidence intervals (CIs) using exact Pearson–Clopper limits. Descriptive statistics were used to summarise AEs for the safety set.

SAS® version 9.4 was used in all the statistical analyses.

## Results

### Patients

A total of 60 patients (21 females) were enrolled between April 19, 2012 and May 02, 2014. Of these, 25 and 35 patients were included in the dose-escalation and dose-expansion part, respectively. The baseline characteristics and demographics of patients are described in Table 1. The median age of the patients was 56.5 years; 65% were male and 93.3% were Caucasian, and 48 patients (80%) had undergone complete gross resection of primary GIST.

**Table 1:** Baseline characteristics and demographics (FAS).

Parameters	Buparlisib daily dose (+imatinib 400 mg)					All patients
	40 mg *n* = 4	50 mg *n* = 4	70 mg *n* = 3	80 mg *n* = 43	100 mg *n* = 6	*n* = 60
Age (years)						
Median (range)	40.5 (30–72)	52.0 (44–63)	63.0 (56–78)	57.0 (28–78)	63.5 (42–72)	56.5 (28–78)
Age category (years), *n* (%)						
<65	3 (75.0)	4 (100)	2 (66.7)	31 (72.1)	3 (50.0)	43 (71.7)
≥65	1 (25.0)	0	1 (33.3)	12 (27.9)	3 (50.0)	17 (28.3)
Sex, *n* (%)						
Male	2 (50.0)	2 (50.0)	3 (100)	27 (62.8)	5 (83.3)	39 (65.0)
Female	2 (50.0)	2 (50.0)	0	16 (37.2)	1 (16.7)	21 (35.0)
Race, *n* (%)						
Caucasian	4 (100)	4 (100)	3 (100)	39 (90.7)	6 (100)	56 (93.3)
Asian	0	0	0	3 (7.0)	0	3 (5.0)
Black	0	0	0	1 (2.3)	0	1 (1.7)
ECOG performance status, *n* (%)						
0	0	2 (50.0)	3 (100)	24 (55.8)	4 (66.7)	33 (55.0)
1	4 (100)	2 (50.0)	0	18 (41.9)	2 (33.3)	26 (43.3)
2	0	0	0	1 (2.3)	0	1 (1.7)
Mitotic index (per 50 HPFs)^a^						
≤5	0	1 (25.0)	0	9 (20.9)	1 (16.7)	11 (18.3)
>5 to ≤10	0	0	0	5 (11.6)	1 (16.7)	6 (10.0)
>10	0	3 (75.0)	1 (33.3)	8 (18.6)	2 (33.3)	14 (23.3)
Missing	2 (50.0)	0	2 (66.7)	13 (30.2)	2 (33.3)	19 (31.7)
Complete gross resection (primary tumour)						
No	2 (50.0)	0	0	10 (23.3)	0	12 (20.0)
Yes	2 (50.0)	4 (100)	3 (100)	33 (76.7)	6 (100)	48 (80.0)
Number of prior regimens						
2	2 (50.0)	3 (75.0)	0	12 (27.9)	3 (50.0)	20 (33.3)
3	1 (25.0)	0	1 (33.3)	24 (55.8)	0	26 (43.3)
4	0	0	1 (33.3)	6 (14.0)	1 (16.7)	8 (13.3)
≥5	1 (25.0)	1 (25.0)	1 (33.3)	1 (2.3)	2 (33.3)	6 (10.0)
Time since initial diagnosis (primary site) to the first dose of study treatment, median (range), months	68.0 (42.6–305.1)	68.3 (56.5–79.3)	85.9 (74.7–128.9)	67.6 (6.2–181.6)	65.8 (40.6–124.9)	70.4 (6.2–305.1)

*ECOG* Eastern Cooperative Oncology Group, *FAS* full-analysis set, *HPF* high-power field.

^a^If the mitotic count was provided for HPFs other than 50, the count was scaled by multiplying it by 50/number of HPF used.

Forty-seven patients (78.3%) discontinued due to progressive disease, 10 (16.7%) due to AEs and 3 (5%) due to patient’s choice.

### Treatment exposure

Overall, the median duration of exposure to study treatment was 2.2 months (range, 0.2–36.8); over 50% of the patients received treatment for more than 2 months, and 20% of the patients received treatment for more than 6 months. Thirty-nine patients (65.0%) had at least 1 interruption or dose change of buparlisib, including 1 patient with dose escalation, and 33 patients (55.0%) had at least 1 interruption/dose reduction of imatinib.

### Maximum tolerated dose

The dose-determining set consisted of 25 patients over 5 dose-level cohorts of buparlisib along with 400 mg of imatinib. In cohort 1 (40 mg of buparlisib, *n* = 4), none of the patients reported dose-limiting toxicities (DLTs); in cohort 2 (50 mg of buparlisib, *n* = 4), one patient reported a DLT of anaphylaxis. Cohorts 3 (70 mg, *n* = 3) and 4 (80 mg of buparlisib, *n* = 4) reported no DLTs, and in cohort 5 (100 mg of buparlisib, *n* = 6), three patients experienced DLTs (stomatitis, hyperglycaemia and depression). Consequently, four additional patients were treated at the previously tested dose level (80 mg of buparlisib and 400 mg of imatinib), and none of them experienced a DLT. The MTD for buparlisib in combination with 400 mg of imatinib was therefore declared as 80 mg.

### Efficacy

The analysis of efficacy was performed on the patients in the dose-expansion part (*n* = 35). All patients received 80 mg of buparlisib in combination with 400 mg of imatinib. A best overall response of SD was observed in 19 patients (54.3%). None of the patients achieved a CR or PR (Table 2). The DCR was 54.3% (95% CI, 36.6–71.2), and the CBR was 28.6% (95% CI, 14.6–46.3). The estimated median PFS was 3.5 months (95% CI, 1.9–5.4), and the estimated PFS rate (Table 3) at 6 months was 22.4% (95% CI, 9.4–38.7).

**Table 2:** The best overall response (FAS, dose-expansion part).

Parameters	All patients *n* = 35, n (%)	95% CI
Best overall response		
Complete response (CR)	0	–
Partial response (PR)	0	–
Stable disease (SD)	19 (54.3)	–
Progressive disease (PD)	14 (40.0)	–
Unknown	2 (5.7)	–
Clinical benefit rate (CBR): CR + PR + SD ≥ 16 weeks	10 (28.6)	14.6–46.3
Overall response rate (ORR): CR + PR	0	0–10.0
Disease control rate (DCR): CR + PR + SD	19 (54.3)	36.6–71.2

*CI* confidence interval, *FAS* full-analysis set.

**Table 3:** Analysis of progression-free survival using Kaplan–Meier method (FAS, dose-expansion part).

	All patients *n* = 35
Number of events, *n* (%)	29 (82.9)
Progression	27 (77.1)
Death	2 (5.7)
Number of censoring, n (%)	6 (17.1)
Percentiles (95% CI) (months)	
25th	1.9 (1.7–1.9)
50th	3.5 (1.9–5.4)
75th	5.5 (3.7–11.4)
% Event-free probability estimates (95% CI)	
2 months	57.4 (38.8–72.2)
4 months	37.3 (20.8–53.8)
6 months	22.4 (9.4–38.7)
8 months	18.6 (7.0–34.6)
11 months	11.2 (2.9–25.8)

*CI* confidence interval, *FAS* full-analysis set.

### Safety

All patients had experienced at least one AE, and 39 patients (65.0%) experienced AEs of grade 3 or 4. The most common all-grade AEs, regardless of the relationship to study treatment in the overall population, were nausea (63.3%), fatigue (48.3%), diarrhoea (45.0%), decreased appetite, hyperglycaemia (28.3% each), abdominal pain and anaemia (25.0% each). The most common grade 3 or 4 AEs included rash (6.7%), diarrhoea, hypophosphataemia, asthenia, rash maculopapular, hypokalaemia and decreased appetite (5.0% each). Overall, 98.3% of patients had AEs suspected to be related to the study drug; 45.0% of those reported grade 3 or 4 AEs. The most common AEs suspected to be drug related (Table 4) were nausea (48.3%) and fatigue (38.3%). Thirteen patients (21.7%) reported AEs, leading to discontinuation of study treatment (Table 5). In nine patients, these AEs were suspected to be related to the study drug, and in seven patients, these were grade 3 or 4 AEs. The main AE leading to discontinuation was depression (three patients).

**Table 4:** Adverse events suspected to be treatment related in ≥15% of patients (safety set).

AEs	Buparlisib daily dose (+imatinib 400 mg)										All patients	
	40 mg *n* = 4, *n* (%)		50 mg *n* = 4, *n* (%)		70 mg *n* = 3, *n* (%)		80 mg *n* = 43, *n* (%)		100 mg *n* = 6, *n* (%)		*n* = 60, *n* (%)	
	All grades	Grade 3 or 4	All grades	Grade 3 or 4	All grades	Grade 3 or 4	All grades	Grade 3 or 4	All grades	Grade 3 or 4	All grades	Grade 3 or 4
Total	4 (100)	1 (25.0)	4 (100)	1 (25.0)	3 (100)	1 (33.3)	42 (97.7)	20 (46.5)	6 (100)	4 (66.7)	59 (98.3)	27 (45.0)
Nausea	0	0	3 (75.0)	0	1 (33.3)	0	22 (51.2)	1 (2.3)	3 (50.0)	0	29 (48.3)	1 (1.7)
Fatigue	1 (25.0)	0	0	0	2 (66.7)	0	17 (39.5)	0	3 (50.0)	0	23 (38.3)	0
Diarrhoea	2 (50.0)	0	0	0	1 (33.3)	0	16 (37.2)	1 (2.3)	2 (33.3)	1 (16.7)	21 (35.0)	2 (3.3)
Hyperglycaemia	0	0	1 (25.0)	0	1 (33.3)	0	9 (20.9)	0	5 (83.3)	1 (16.7)	16 (26.7)	1 (1.7)
Rash	0	0	0	0	1 (33.3)	0	11 (25.6)	3 (7.0)	1 (16.7)	1 (16.7)	13 (21.7)	4 (6.7)
Decreased appetite	1 (25.0)	1 (25.0)	1 (25.0)	0	0	0	6 (14.0)	1 (2.3)	2 (33.3)	0	10 (16.7)	2 (3.3)

*AE* adverse event.

**Table 5:** Summary of deaths, serious adverse events and adverse events leading to discontinuations (safety set).

	Buparlisib daily dose (+ imatinib 400 mg)										All patients	
	40 mg		50 mg		70 mg		80 mg		100 mg			
	*n* = 4, *n* (%)		*n* = 4, *n* (%)		*n* = 3, *n* (%)		*n* = 43, *n* (%)		*n* = 6, *n* (%)		*n* = 60, *n* (%)	
	All grades	Grade 3 or 4	All grades	Grade 3 or 4	All grades	Grade 3 or 4	All grades	Grade 3 or 4	All grades	Grade 3 or 4	All grades	Grade 3 or 4
All deaths^a^	2 (50.0)	–	1 (25.0)	–	0	–	7 (16.3)	–	2 (33.3)	–	12 (20.0)	–
On-treatment deaths^b^	0	–	0	–	0	–	3 (7.0)	–	0	–	3 (5.0)	–
AEs	4 (100)	2 (50.0)	4 (100)	2 (50.0)	3 (100)	1 (33.3)	43 (100)	29 (67.4)	6 (100)	5 (83.3)	60 (100)	39 (65.0)
Suspected to be drug related	4 (100)	1 (25.0)	4 (100)	1 (25.0)	3 (100)	1 (33.3)	42 (97.7)	20 (46.5)	6 (100)	4 (66.7)	59 (98.3)	27 (45.0)
SAEs	1 (25.0)	1 (25.0)	2 (50.0)	2 (50.0)	1 (33.3)	0	16 (37.2)	15 (34.9)	3 (50.0)	3 (50.0)	23 (38.3)	21 (35.0)
Suspected to be drug related	0	0	1 (25.0)	1 (25.0)	0	0	5 (11.6)	5 (11.6)	2 (33.3)	2 (33.3)	8 (13.3)	8 (13.3)
AEs leading to discontinuation	0	0	1 (25.0)	1 (25.0)	1 (33.3)	0	9 (20.9)	6 (14.0)	2 (33.3)	2 (33.3)	13 (21.7)	9 (15.0)
Suspected to be drug related	0	0	1 (25.0)	1 (25.0)	0	0	6 (14.0)	4 (9.3)	2 (33.3)	2 (33.3)	9 (15.0)	7 (11.7)
AEs requiring dose interruption and/or change	1 (25.0)	1 (25.0)	1 (25.0)	1 (25.0)	1 (33.3)	1 (33.3)	32 (74.4)	17 (39.5)	3 (50.0)	1 (16.7)	38 (63.3)	21 (35.0)
Suspected to be drug related	0	0	0	0	1 (33.3)	1 (33.3)	28 (65.1)	14 (32.6)	3 (50.0)	1 (16.7)	32 (53.3)	16 (26.7)
AEs requiring additional therapy	4 (100)	1 (25.0)	4 (100)	2 (50.0)	3 (100)	1 (33.3)	41 (95.3)	22 (51.2)	6 (100)	3 (50.0)	58 (96.7)	29 (48.3)
Suspected to be drug related	3 (75.0)	1 (25.0)	3 (75.0)	1 (25.0)	1 (33.3)	1 (33.3)	37 (86.0)	13 (30.2)	5 (83.3)	2 (33.3)	49 (81.7)	18 (30.0)

*AE* adverse event, *SAE* serious adverse event.

^a^All deaths, including those >30 days after the end of treatment.

^b^Deaths occurring >30 days after the end of treatment are not included.

### Serious adverse events

In total, 23 patients (38.3%) experienced serious AEs (SAEs, Table 5). The most common SAEs were abdominal pain, hyponatraemia, nausea and peritoneal haemorrhage in two patients each (3.3%). Thirteen SAEs were suspected to be drug related in eight patients (13.3%), and included anaphylactic reaction, hypokalaemia, hypophosphataemia, nausea, dementia, rash maculopapular, posterior reversible encephalopathy syndrome, hyponatraemia, depression, anxiety, delirium, rash and hyperglycaemia. Of these eight patients, four permanently discontinued the study treatment due to SAEs pertaining to depression, anxiety/delirium/rash/hyperglycaemia, posterior reversible encephalopathy syndrome and anaphylactic reaction, respectively.

### Neuropsychiatric AEs

In total, 25 patients (41.7%) experienced AEs related to psychiatric disorders, of whom, 18 (30.0%) had drug-related AEs. The most common psychiatric disorders regardless of the relationship to study treatment (≥3% of patients) were insomnia in 12 (20.0%), anxiety in 9 (15.0%), depression in 8 (13.3%), irritability in 3 (5.0%) and depressed and altered mood in 2 patients each (3.3%). Three patients (5.0%) reported grade 3 or 4 AEs of insomnia, depression and hallucination, which were all suspected to be related to the study drug as per the investigator. Four patients reported psychiatric disorders leading to treatment discontinuation; none of them were due to mood disorders. The treatment was temporarily interrupted in seven patients due to psychiatric disorders, and in two patients due to mood disorders (one each for euphoric and depressive mood).

### Deaths

Overall, 12 (20.0%) deaths were reported (Table 5). This included three on-treatment deaths (two due to GIST and one due to infectious meningitis), defined as either death on treatment or death within 30 days of the last dose. Seven patients died after the treatment discontinuation due to GIST, and two patients due to unknown reasons. The on-treatment death due to AEs (meningitis) was not considered to be related to the study treatment by the investigator.

## Discussion

A major challenge in the treatment of GIST is resistance to TKIs, which can be primary resistance, or more commonly due to the acquisition of secondary *KIT/PDGFRA* mutations or activation of alternative signalling pathways, such as the PI3K/AKT/mTOR pathway.^23^ Another mechanism of resistance to targeted therapies is due to insufficient levels of phosphatase and tensin homologue (PTEN) protein, which negatively regulates the PI3K/AKT signalling pathway.^24^ The PI3K/AKT pathway plays an important role in the proliferation and survival of imatinib-sensitive and -resistant GIST.^25^ Abrogation of the PI3K-binding site in murine models with *KIT* mutations prevented the development of GIST.^26^ Buparlisib is a pan-PI3K inhibitor that targets all the isoforms of class I PI3K, including PI3Kδ, which was hypothesised to play an important role in imatinib-resistant GIST.^27^ One of the most promising approaches is to inhibit the PI3K pathway in addition to continuous inhibition of *KIT/PDGFRA* mutations by imatinib. This concept had been validated in patient-derived mouse xenografts of GIST. Single-agent buparlisib was able to reduce tumour volume in mice grafted with human GIST carrying diverse *KIT* genotypes and PTEN genomic status, and a greater reduction was observed in combination with imatinib.^20^ The activity of GDC-0941, another PI3K inhibitor, in combination with imatinib, was also reported in a murine model of GIST, with a concurrent loss of the protein and gene expression of PTEN.^25^ In preclinical studies with buparlisib, better efficacy was observed with the combination of GDC-0941 and imatinib compared with single agent alone, with a reduction in tumour growth even after discontinuation of the drug. Similar results were also emulated in preclinical studies with the combination of an AKT inhibitor MK-2206 and imatinib in GIST xenografts,^28^ thus highlighting the importance of PI3K inhibitors in advanced GIST.

Imatinib is approved as a first-line therapy for the treatment of unresectable and/or metastatic GIST, and has shown a PFS of up to 24 months and an overall survival of up to 57 months.^29,30^ Adjuvant imatinib has also shown clinical benefits, including a significant improvement in recurrence-free survival, and 400 mg of imatinib is also approved as adjuvant therapy.^10,31^ Rechallenge with imatinib in patients with metastatic and/or unresectable GIST following objective progression after treatment failure with imatinib and sunitinib has shown encouraging results. The RIGHT trial randomised patients with progressive disease following imatinib/sunitinib with or without other agents, to receive either imatinib rechallenge or placebo (ClinicalTrials.gov, ID No. NCT01151852).^32^ This trial reported a median PFS of 1.8 months in the imatinib rechallenge arm and 0.9 months in the placebo arm. The BFR14 study conducted by the French Sarcoma group also demonstrated the efficacy of imatinib rechallenge in patients who progressed after imatinib discontinuation, and more than 90% of the patients achieved a tumour response.^33^ In another retrospective trial conducted in Italy in patients with advanced GIST who were previously treated with imatinib, sunitinib and regorafenib, rechallenge with imatinib resulted in a median time to progression of 5.4 months.^34^ This further suggested that rechallenge with imatinib could be an option for patients with advanced GIST. The current study demonstrated an estimated median PFS of 3.5 months (95% CI, 1.9–5.4) with the combination of buparlisib and imatinib. The results of this Phase 1 trial of buparlisib and imatinib compare favourably with those of the RIGHT trial, although a direct comparison in these small groups is not possible.^32^

Regorafenib is an oral multi-targeted inhibitor with activity against multiple kinases, including KIT, RET, RAF1 and BRAF, as well as those involved in angiogenesis (VEGFR and TIE-2) and in tumour support from the microenvironment (PDGFR and FGFR).^35^ The GRID study, a randomised, Phase 3 trial with regorafenib monotherapy in progressive GIST, reported a median PFS of 4.8 months (hazard ratio, 0.27; 95% CI, 0.19–0.39; *P* < 0.0001) when treated with the active compound in the third line.^36^ Regorafenib was shown to significantly improve PFS and DCR compared with placebo. In an earlier Phase 2 trial with 34 patients, treatment with third-line regorafenib showed a PFS of 10 months.^37^ The current study and both the GRID and RIGHT studies included patients with advanced GIST who had progressed after failure of at least imatinib and sunitinib.^32,36^ Another study of imatinib in combination with everolimus (targeting the PI3K/AKT/mTOR pathway) reported a median PFS of 3.5 months (95% CI, 1.9–5.2) in patients who failed imatinib and sunitinib therapies.^38^ Although preclinical studies suggested that the combination of a PI3K inhibitor with imatinib may be beneficial for these patients,^20^ in this study, buparlisib demonstrated no objective responses and limited DCR with a median treatment exposure of 2.2 months, in combination with imatinib, and a few patients had durable SD. The results from this study may also need to be assessed, considering the relatively good Eastern Cooperative Oncology Group (ECOG) performance status (98% with ECOG 0 or 1) and slightly lower median age (56.5 years) of the patients enrolled into this study compared with the other studies mentioned, and to patients treated in clinical practice.^9,32,34,39^ Although the study included patients with an ECOG performance status between 0 and 2 considering other criteria for inclusion in the study, it is possible that more patients with a better performance status were included. Although the median age was slightly lower at 56.5 years than that of the other studies, which was mostly around 60 years, meaningful comparisons cannot be drawn between populations across clinical trials as the population age range is overlapping.^9,32,34,39^ Hence, there remains a high unmet medical need in the management of advanced GIST, as highlighted by a median PFS of 4.8 months for regorafenib in the randomised Phase 3 trial, indicating that durable clinical benefit to later lines of therapy remains elusive.^36^

For metastatic GIST, imatinib was used in doses ranging from 400 to 800 mg, and a higher PFS was observed with the 800-mg dose in GIST with *KIT* exon 9 mutations, although the number of interruptions were more.^30,40^ In a Phase 1, dose-finding study of buparlisib in patients with advanced solid tumours, the MTD was determined to be 100 mg.^41^ In this study, the MTD was 80 mg for buparlisib in combination with 400 mg for imatinib, which was 80% of the MTD of buparlisib as monotherapy (confirmed in Phase 1 studies in patients with solid tumours).

Buparlisib has a toxicity profile, which although not negligible, is manageable, with AEs mostly related to gastrointestinal disorders, hyperglycaemia and mood disorders.^42^ In combination with imatinib, the safety profile was consistent but manageable. As compared with the current standard of care regorafenib, in the GRID trials, the severity of the toxicities was similar as almost 98.5% of the patients treated with regorafenib experienced an AE of which 58.3% experienced an AE of grade 3 and above, although the specific toxicities of the two molecules were different.^36^ Hypertension and hand–foot skin reaction were the most common drug-related AEs with regorafenib of whom ~20% of the patients experienced grade 3 AEs. The neuropsychiatric AEs were consistent with the previous reports, and could be attributed to the high blood–brain barrier–penetration properties of buparlisib.^19,43^ In the preclinical studies in rodents, buparlisib was able to penetrate the blood–brain barrier and subsequently downregulate the PI3K pathway in the brain of animals.

Although the current study demonstrated slightly better PFS than prior reports of imatinib rechallenge in a resistant population, there was no clear efficacy signal to expect improved clinical outcomes in the context of currently approved therapies for GIST.^44^ Given the limited activity, the benefit–risk balance does not favour the use of this combination in patients who have failed prior therapy with imatinib and sunitinib. Further development of this combination therapy of buparlisib with 400 mg of imatinib is not recommended in the third-line/fourth-line treatment of patients with advanced/metastatic GIST. It would be, however, interesting to further explore this pathway to identify predictors of tumour control under such combination.

## Supplementary information

IRB centers

## Data Availability

Novartis will not provide access to patient-level data, if there is a reasonable likelihood that individual patients could be re-identified. Phase 1 studies, by their nature, present a high risk of patient re-identification; therefore, patient individual results for Phase 1 studies cannot be shared. In addition, clinical data, in some cases, have been collected subject to contractual or consent provisions that prohibit transfer to third parties. Such restrictions may preclude granting access under these provisions. Where co-development agreements or other legal restrictions prevent companies from sharing particular data, companies will work with qualified requestors to provide summary information where possible.
