# Supplementary material for: Imatinib in combination with phosphoinositol kinase inhibitor buparlisib in patients with gastrointestinal stromal tumour who failed prior therapy with imatinib and sunitinib: a Phase 1b, multicentre study
Source: Br J Cancer. 2020 Mar 9;122(8):1158–65. doi: 10.1038/s41416-020-0769-y (PMC7156686; doi:10.1038/s41416-020-0769-y)
Supplement: Supplementary file 1 — IRB centers [file 41416_2020_769_MOESM1_ESM.pdf]

1 List of Independent Ethics Committees (IECs) or Institutional Review Boards (IRBs)

Table 1-1 List of Independent Ethics Committees (IEC) or Institutional Review Boards (IRB) by study center

Center No.	Ethics Committee or Institutional Review Board	Department / Organization	City, State/Province, Postal Code Country
1000	Commissie Medische Ethiek Van De Universitaire Ziekenhuizen Kuleuven		Leuven B-3000 Belgium
2000	UBC BCCA Research Ethics Board	BC Cancer Agency	Vancouver BC V5Z 1H8 Canada
9000	Comitè Ètic d'Investigació Clinica	Clinica de l'Hospital Universitari Vall d'Hebron	Barcelona Cataluna 08035 Spain
3001	CPP (Comite' de Protection des Personnes)	Centre Léon Bérard	Lyon Cedex 08 69373 France
3002	CPP (Comite' de Protection des Personnes)	Centre Léon Bérard	Lyon Cedex 08 69373 France
8000	NHS Health Research Authority - NRES Committee London - West London		London W6 8RF United Kingdom
8001	NHS Health Research Authority - NRES Committee London - West London		London W6 8RF United Kingdom
2500	National Cancer Center IRB		Chiba and Tokyo 277-8577 and 104-0045 Japan
7000	LUMC (Leids Universitair Medisch Centrum)		Leiden 2333 ZA Netherlands
5001	Dana-Farber Cancer Institute Institutional Review Board		Boston MA 02215 USA
5003	Western Institutional Review Board		Puyallup WA 98374 USA
